# Implementation of Cell Samples as Controls in National Proficiency Testing for Clopidogrel Therapy-Related *CYP2C19* Genotyping in China: A Novel Approach

**DOI:** 10.1371/journal.pone.0134174

**Published:** 2015-07-28

**Authors:** Guigao Lin, Lang Yi, Kuo Zhang, Lunan Wang, Rui Zhang, Jiehong Xie, Jinming Li

**Affiliations:** 1 National Center for Clinical Laboratories, Beijing Hospital, Beijing 100730, P R China; 2 Graduate School, Peking Union Medical College, Chinese Academy of Medical Sciences, Beijing 100730, P R China; IPO, Portuguese Institute of Oncology of Porto, PORTUGAL

## Abstract

Laboratories are increasingly requested to perform *CYP2C19* genetic testing when managing clopidogrel therapy, especially in patients with acute coronary syndrome undergoing percutaneous coronary intervention. To ensure high quality molecular testing and ascertain that the referring clinician has the correct information for *CYP2C19* genotype–directed antiplatelet therapy, a proficiency testing scheme was set up to evaluate the laboratory performance for the entire testing process. Proficiency panels of 10 cell samples encompassing the common *CYP2C19* genetic polymorphisms were distributed to 62 participating laboratories for routine molecular testing and the responses were analyzed for accuracy of genotyping and the reporting of results. Data including the number of samples tested, the accreditation/certification status, and test methodology of each individual laboratory were also reviewed. Fifty-seven of the 62 participants correctly identified the *CYP2C19* variants in all samples. There were six genotyping errors, with a corresponding analytical sensitivity of 98.5% (333/338 challenges; 95% confidence interval: 96.5–99.5%) and an analytic specificity of 99.6% (281/282; 95% confidence interval: 98.0–99.9%). Reports of the *CYP2C19* genotyping results often lacked essential information. In conclusion, clinical laboratories demonstrated good analytical sensitivity and specificity; however, the pharmacogenetic testing community requires additional education regarding the correct reporting of *CYP2C19* genetic test results.

## Introduction

Clopidogrel irreversibly inhibits the P2RY12 receptor on platelets and reduces the rate of major vascular events in patients with acute coronary syndromes undergoing percutaneous coronary intervention (PCI) [1]. Clopidogrel is a prodrug and cytochrome P450 (CYP) isoenzymes, particularly CYP2C19, are required for the conversion of clopidogrel to its active metabolite [2]. Polymorphisms in *CYP2C19* have been consistently shown to be associated with the interindividual variability of response to clopidogrel [3]. Patients harboring two loss-of-function *CYP2C19* alleles (including the *2 and *3 alleles) have lower levels of active clopidogrel metabolites, diminished platelet inhibition and an increased risk of major adverse cardiovascular events [4–6]. Unlike other CYP450s, *CYP2C19* *2(c.681G>A; rs4244285) occurs more frequently in Asians (29–35%) than in Caucasians and Africans (~15%) [7], and the *CYP2C19* *3(c.636G>A; rs4986893) allele frequency is also higher in Asians (with an allele frequency of 2–9%) than in other ethnic groups (typically below 1%) [7]. Several studies suggested that carriers of the CYP2C19*17 allele (c.-806C>T; rs12248560) have higher antiplatelet responses and a possibly increased bleeding risk [8–9]. However, other studies did not detect an association [10–11].

Based on the early findings and related pharmacokinetic and pharmacodynamic data, the US Food and Drug Administration issued a black box warning of reduced effectiveness of clopidogrel in carriers of two loss-of-function alleles. Since genetic testing is increasingly widespread, the Clinical Pharmacogenetics Implementation Consortium provided guidelines for clopidogrel therapy in acute coronary syndromes/PCI patients whose *CYP2C19* genetic information is available [7]. Alternative therapy is recommended for patients carrying a *CYP2C19* loss-of-function allele.

With strong evidence in the literature to suggest the potential benefit of guiding clopidogrel therapy for patients undergoing PCI, the implementation of clopidogrel pharmacogenetic testing is becoming increasingly common in clinical laboratories. However, not all of them have devoted sufficient attention to issues of quality control. The success of genotype-guided clopidogrel therapy would be expected to be largely dependent on the reliability of testing; thus, the Chinese National Center for Clinical Laboratories (NCCL) conducted a proficiency test (PT) in 2015 to evaluate the performance of clopidogrel related genetic testing, including the correct identification of *CYP2C19* genotypes, and reporting of test results. Cell samples instead of DNA were used in order to assess the pre-analytical process. This report provides a comprehensive picture of the accuracy and reliability of *CYP2C19* testing in China.

## Materials and Methods

### Organization of the scheme

Laboratories performing analysis of *CYP2C19* polymorphisms were invited to participate in this quality control scheme. No criteria were set for participation and a total of 62 laboratories participated in the national *CYP2C19* PT scheme in 2015. We used cell samples to evaluate the DNA extraction process in this PT survey. Table 1 summarizes the composition of the *CYP2C19* PT panel (n = 10). The participants were required to perform DNA extraction and genotyping in their routine procedures. Cell samples were delivered at ambient temperature and the laboratories were advised to evaluate and process the samples immediately on receipt. Submission of results had to be within 10 days of receiving the samples. Participating laboratories were asked to provide a genotype result for each sample. The laboratories were also requested to offer information about the genotyping method employed, the number of tests for *CYP2C19* polymorphisms performed each month, and the laboratory accreditation/certification status.

**Table 1:** PT panel and the results of genotyping accuracy for the 2015 NCCL/*CYP2C19* proficiency testing survey.

Sample	Coriell Cell Line Number	Expected genotype ^a^	No. Correct/challenges ^b^	No. Correct/Total challenges	Accuracy, %	No. error
CU1501	GM17248	*1/*1(*17/*17)	47/47(15/15)	62/62	100	0
CU1502	GM17220	*1/*1(*1/*17)	46/47(15/15)	61/62	98.3	1
CU1503	GM16688	*2/*3	46/47(15/15)	61/62	98.3	1
CU1504	GM17289	*2/*2	47/47(15/15)	62/62	100	0
CU1505	GM17052	*1/*3	46/47(15/15)	61/62	98.3	1
CU1506	GM17260	*1/*2(*2/*17)	47/47(14/15)	61/62	98.3	1
CU1507	GM17285	*1/*1	47/47(15/15)	62/62	100	0
CU1508	GM17285	*1/*1	47/47(15/15)	62/62	100	0
CU1509	GM17285	*1/*1	47/47(15/15)	62/62	100	0
CU1510	GM17260	*1/*2(*2/*17)	46/47(14/15)	60/62	96.7	2

^a^ Genotypes in parentheses would be reported by laboratories which detect the CYP2C19*17 allele.

^b^ Fifteen laboratories screened for the CYP2C19*17 allele.

### Preparation of PT panel

Cell lines (Table 1) with common *CYP2C19* genetic polymorphisms were purchased from The National Institute of General Medical Sciences Repository at the Coriell Cell Repositories (Coriell, New Jersey, USA). The characterization of these cell lines for *CYP2C19* polymorphisms had been determined by using a variety of assay platforms [12]. The cells were cultured in Roswell Park Memorial Institute 1640 supplemented with 5% fetal bovine serum, 2mM L-glutamine, 10U/mL penicillin and 10μg/mL streptomycin (Invitrogen, Carlsbad, USA). On the sample delivery day, cells were collected and resuspended in fresh medium at a concentration of 1×10^6^ cells/mL. One thousand microliters of each sample was dispensed in 1.5-mL vials and labeled.

### Validation of PT panel

The NCCL reference lab validated the samples before shipping them to participants. Sanger sequencing was performed on all the cell lines to verify the genotype. Genomic DNA was extracted using the QIAamp DNA Mini Kit (QIAGEN, Hilden, Germany). The specific primers were used to amplify the sequences of the *CYP2C19* gene containing the three single nucleotide polymorphism (*2, *3, *17). Sequencing reactions were done with purified polymerase chain reaction (PCR) products by using BigDye Terminator v3.1 Cycle Sequencing Kit (Applied Biosystems, Foster City, USA) and the sequence analysis was performed by using ABI 3500DX Genetic Analyzer (Applied Biosystems) according to the manufacturer's instructions. All sequencing results were analyzed with Chromas software and were verified by manual inspection. To simulate transport conditions, cell samples were incubated at room temperature for one week and then analyzed.

### Data analysis

The submitted results were analyzed by NCCL experts and the criterion used for considering a data set proficient is at least 80% genotype accuracy.

Genotyping accuracy, types of errors, analytical sensitivity, and specificity were computed and a comparison of the rates was performed by Fisher’s exact test with two-tailed statistical significance at P < 0.05. Confidence intervals of 95% (CI 95%) were determined. All data analyses were performed by using the MEDCALC software program (MedCalc Software, Mariakerke, Belgium).

### Scoring of the reports

Each laboratory was asked to send a written report of the results for the first sample (CU1501). The assessment of the reports was used for educational purposes only and was not graded. According to the International Organization for Standardization (ISO) 15189:2007 requirements for medical laboratories [13], we defined 16 key items that should be present in a good report for *CYP2C19* genotyping (Table 2). Each item scored one point if included correctly.

**Table 2:** Different items used for scoring of reports in the 2015 NCCL/*CYP2C19* proficiency testing survey.

Item	Description
1	Sampling/arrival date
2	Sample number
3	Date of report
4	Signature
5	Unique identifier on each page
	• For example, by lab identifier, name. . .
6	Total pages
	• Page 1 of 2, 1/2 (not 1,2,3,. . .)
7	Consultants• Lab address and phone number
8	Nature of the sample
	• Peripheral blood,. . .
9	Reason for testing
10	Genotype
11	Phenotype
12	Interpretation of the results
	• Comments/results and conclusion,. . .
13	List of alleles tested
14	Method used
15	Report title
	• Refers to *CYP2C19* genotyping and is clearly distinguished from other reports
16	Refers to therapy
	•Dosing recommendations,. . .

## Results

### Sample validation

The expected genotype (data from Coriell) of each cell sample was confirmed by Sanger sequencing designed to detect the alleles (Table 1). For laboratories that did not screen for the increased function CYP2C19*17 allele, this allele was listed as *1. A stability study showed that the amount of DNA (>10μg) extracted from each cell sample was adequate for downstream analysis even after incubation for seven days.

### Study population and response

A total of 62 participants, including 48 hospital laboratories, 13 commercial laboratories and 1 reagent manufacturer, submitted the genotyping results within the specified deadline. Of the 62 participating laboratories, 61 labs offered tests for clinical use, 13 laboratories were accredited according to ISO 15189 or ISO 17025 and another 3 laboratories were certified according to the College of American Pathologists (CAP). The mean number of samples tested per month was 47 (range: 1–500, according to the statements in the questionnaire). Twenty-five laboratories analyzed less than ten samples per month.

The most frequently used methodology was pyrosequencing [23/62, (37.0%)], followed by PCR-microarray [21/62, (33.8%)], Sanger sequencing [6/62, (9.6%)], real-time PCR [5/62, (8.0%)], next-generation sequencing (NGS) [3/62, (4.8%)], high-resolution melting analysis (HRMA) [2/62, (3.2%)], matrix-assisted laser desorption/ionization mass spectrometry (MALDI-TOF-MS) [1/62, (1.6%)], and digital florescence in situ hybridization [1/62, (1.6%)]. Forty-nine laboratories applied commercial kits and 13 participants used laboratory-developed tests. Due to the low allele frequency (0.5%-3.0%) for CYP2C19*17 among the Chinese population [14–16], 47 of the participating laboratories did not screen for this gain-of-function allele.

The PT samples were delivered to laboratories across the country within seven days. None of the participants reported DNA extraction problems with the cell samples.

### Performance of participating laboratories and CYP2C19 genotyping assays

Results from the participants were compared with the genotypes that they were expected to detect (Table 1). In total, fifty-seven (91.9%) participants correctly identified all of the ten PT challenges (100% proficient), four laboratories made one genotype mistake (90% proficient), and one laboratory made two mistakes (80% proficient). The performances of *CYP2C19* genotyping among the different types of participating groups were compared. There was no statistical difference in genotyping accuracy between hospital laboratories and commercial laboratories *(P* = 0.110), or between accredited and non-accredited laboratories *(P* = 1). It should be noted that the non-accredited laboratories made five mistakes.

Two types of errors were observed: false-positive results (mutation instead of wild type) and false-negative results (not reported and wrong mutation). Table 3 shows the details of the mistakes made by participants in the 2015 NCCL/*CYP2C19* genotyping survey. Among the six errors, two were results not reported. A mutation was present in three instances, but was incorrectly reported. One false positive also occurred where a mutation (c.681G>A) was reported when none was present in the sample.

**Table 3:** The intended genotypes and actual responses for six genotyping errors identified in the 2015 NCCL/*CYP2C19* proficiency testing survey.

Sample	Testing method	Intended response	Actual response	Error type
CU1502	PCR-microarray-BiaO	*1/*1	*1/*2	False positive
CU1503	PCR-microarray-BiaO	*2/*3	*1/*3	False negative
CU1505	In-house Sanger sequencing	*1/*3	Not reported	False negative
CU1506	In-house HRMA	*2/*17	*1/*2	False negative
CU1510	Pyrosequencing-Qiagen	*1/*2	Not reported	False negative
CU1510	In-house HRMA	*2/*17	*1/*2	False negative

We next evaluated the performances of different assays. The proficiency of *CYP2C19* genotyping by assay is shown in Table 4. Fifty-seven data sets were 100% proficient (correct identification of all challenges). Five data sets were 80%–99% proficient. The analytical sensitivity and specificity of testing methods were also determined (Table 4). The overall sensitivity and specificity were both high (98.5% and 99.6%, respectively) in this PT survey; however, one in-house developed assay showed a lower analytical sensitivity (in-house HRMA, 71.4%).

**Table 4:** Proficiency results and characteristics of genotyping methods used in the 2015 NCCL/*CYP2C19* proficiency testing survey.

Assay	No. of data sets	No. of data sets proficient at ^a^:				*CYP2C19* genotypes	
		100%	99–90%	89–80%	< 80%	Sensitivity (%; CI 95%) Correct mutations/total mutant challenges	Specificity (%; CI 95%) Correct wild-types /total wild-type challenges
Pyrosequencing-Qiagen	22	21	1	0	0	99.0; 95.0–99.9 (109/110)	100;96.7–100 (110/110)
In-house Pyrosequencing	1	1	0	0	0	100;47.8–100 (5/5)	100;47.8–100 (5/5)
PCR-microarray-BaiO	19	17	2	0	0	98.9; 94.2–99.9 (94/95)	98.9; 94.2–99.9 (94/95)
In-house PCR-microarray	2	2	0	0	0	100; 76.8–100 (14/14)	100; 54.0–100 (6/6)
Sanger sequencing-Szyt	1	1	0	0	0	100; 59.0–100 (7/7)	100; 29.2–100 (3/3)
In-house Sanger sequencing	5	4	1	0	0	96.7; 83.3- 99.9 (30/31)	100; 82.3–100 (19/19)
Real-time PCR-Skybiotech	3	3	0	0	0	100;78.2–100 (15/15)	100;78.2–100 (15/15)
Realtime PCR-Yzybio	2	2	0	0	0	100; 76.8–100 (14/14)	100; 54.0–100 (6/6)
In-house NGS	3	3	0	0	0	100;83.8–100 (21/21)	100;66.3-100(9/9)
HRMA-Szwz	1	1	0	0	0	100;47.8–100 (5/5)	100;47.8–100 (5/5)
In-house HRMA	1	0	0	1	0	71.4; 29.0- 96.3(5/7)	100;29.2–100 (3/3)
In-house MALDI-TOF-MS	1	1	0	0	0	100; 59.0–100 (7/7)	100; 29.2–100 (3/3)
Digital FISH-Hxsd	1	1	0	0	0	100; 59.0–100 (7/7)	100; 29.2–100 (3/3)
Total	62	57	4	1	0	98.5; 96.5–99. 5(333/338)	99.6;98.0–99.9(281/282)

^a^100% proficient: all genotype detected correctly. 80%–99% proficient: 80%–99% of genotype detected correctly. Not proficient: < 80% of genotype detected correctly.

### Reporting of results

**Fig 1:**
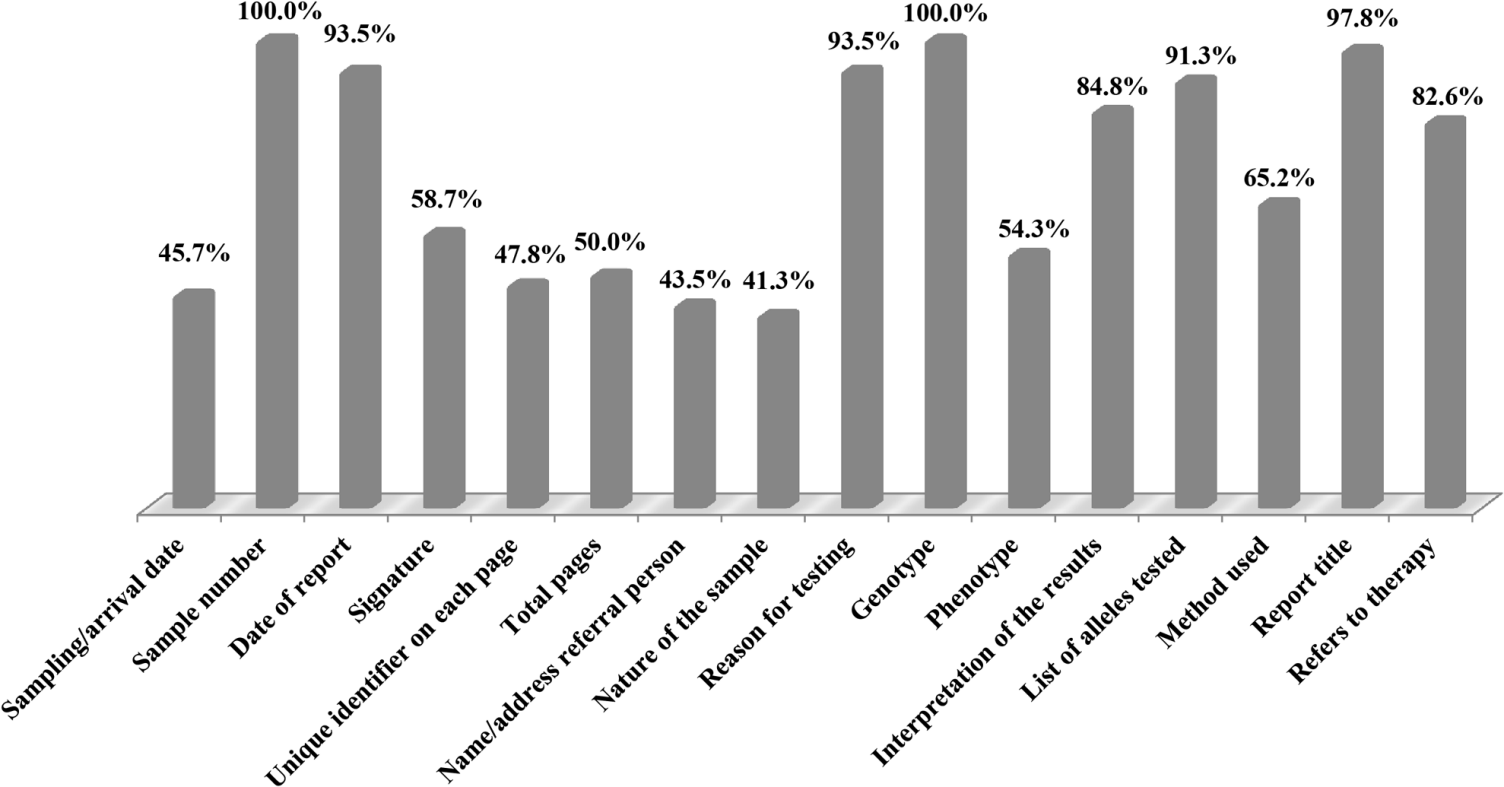
Reporting scores of the 2015 NCCL/*CYP2C19* proficiency testing survey. N = 46 reports analyzed.

In total, 46 of 62 participants submitted reports for the CU1501 sample. The average score of the reports was 11.5 points (out of a maximum of 16). Items that were usually included were sample number, date of report, reason for testing, genotype, interpretation of the results, list of alleles tested, method used, report title and therapy (Fig 1). In contrast, sampling/arrival date, signature, unique identifier on each page, total pages, consultants for the report, nature of the sample, and phenotype were commonly not included in the reports.

## Discussion

The *CYP2C19* enzyme is important for the metabolism of a number of prescription drugs such as amitriptyline, clomipramine, and clopidogrel. Genotyping of three single-nucleotide polymorphisms (*2, *3, *17) that define the major *CYP2C19* alleles is often performed to guide clopidogrel therapy. The purpose of this PT was to assess the performance of laboratories offering *CYP2C19* genetic testing and to enhance the quality of testing by providing a means for education of the pharmacogenetic testing community.

The results of this PT provide evidence of good analytical performance with few genotyping failures. However, any mistakes were considered unacceptable in light of quality control. Of the six genotyping errors found, five were false negatives. For samples CU1505 and CU1510, two labs did not report the genotyping results. Follow-up investigation with the participants showed that one error was due to technical failure, and the other was caused by missing data transcription when submitting the results. For the three cases of mistaken mutations, two were produced by the laboratory that employed an in-house HRMA method. Interestingly, the lab was able to identify the *17 allele in the allele combination of *1/*17 and *17/*17, but failed to detect this allele in the *2/*17 genotype. Thus, it is important for laboratories to validate laboratory-developed mutation tests before using them. The third mistaken mutation may be related to laboratory performance, since the other 17 labs using the same genotyping kit detected all genotypes correctly. One laboratory incorrectly reported CYP2C19*1/*1 as CYP2C19*1/*2. This false-positive genotyping result would mistakenly predict the *CYP2C19* metabolizer phenotype of an individual as an intermediate metabolizer (IM), which may increase the predicted risk for major adverse cardiovascular events (e.g., cardiovascular death, myocardial infarction, stroke, and stent thrombosis) in acute coronary syndromes/PCI patients treated with clopidogrel. Unfortunately, this lab did not provide any further information regarding the cause of the error. False positives can be avoided by adoption of negative controls in a PCR-based assay.

An important issue in *CYP2C19* genotyping is the methodology used for testing. Sanger sequencing of PCR-amplified genomic DNA, which is considered the gold standard, can identify all possible base substitutions. In this study, two commercial testing kits were predominantly used: pyrosequencing-Qiagen (Qiagen) and PCR-microarray-BaiO (BaiO, Shanghai, China). Most of the testing methods which were employed in this PT showed both good analytical sensitivity and specificity. Notably, three participants in this survey used a laboratory-developed test based on NGS without any mistakes. NGS is rapidly incorporated into clinical laboratory methods, increasing technological capacities and decreasing costs. In the field of pharmacogenetics, with complex variants in the different genes associated with individual drug responses, it may be more cost effective to implement exome sequencing in contrast to targeted screening. As NGS-based clinical tests are of much greater complexity than traditional genetic tests, the CAP has developed novel laboratory standards for NGS clinical tests [17].

Another important aspect of clinical pharmacogenetic testing is reporting the results. Reporting of laboratory testing, especially genetic testing, is a necessary step. Complete and accurate reporting is of great important for correctly transferring diagnostic information to the physician requesting the test. Good diagnostic testing is useless if the report provides insufficient or even wrong information. According to the ISO 15189 standard, reporting information on the laboratory, patient, sample identifiers, results, methodology, and interpretation are essential. The overall quality of the reports in this PT survey was less than adequate, and 17 of 61 clinical laboratories did not submit the reports. More education on the reporting of test results is warranted.

A PT scheme provides an objective evaluation of laboratory performance; moreover, it will be of significance to physicians who should appreciate that quality control is very important to produce reliable results in pharmacogenetic testing. Under the circumstances, clinicians should not only be concerned about the testing results, but also about the entire testing process, especially the pre- and post-analytical procedures.

The overall genotype concordance of *CYP2C19* in these PT [614 responses/620 challenges, (99.0%)] is better than that in the CAP pharmacogenetic testing survey [545 responses/603 challenges, (90.3%)] [18]. The main reason is that the accuracy of CYP2C19*1/*17 genotyping in the CAP PT (50.8%) is inferior to our findings in this report (100%).

In conclusion, Chinese laboratories providing a *CYP2C19* genotyping service in the survey had a better analytical performance than reporting performance. **C**ontinued external assessment and the education of reporting genetic results are required, to make sure that the referring physician has accurate information for genotype-guided clopidogrel treatment.
